# Working conditions are associated with the occurrence of sleepiness of nursing professionals: a case-control study

**DOI:** 10.5935/1984-0063.20220042

**Published:** 2022

**Authors:** Maria Carmen Martinez, João Silvestre Silva-Junior, Maria do Rosário Dias de Oliveira Latorre, Frida Marina Fischer

**Affiliations:** 1 WAF Informática e Saúde - São Paulo - SP - Brazil.; 2 Centro Universitário São Camilo, Departamento de Medicina - São Paulo - SP - Brazil.; 3 Universidade de São Paulo, Departamento de Epidemiologia, Faculdade de Saúde Pública - São Paulo - SP - Brazil.; 4 Universidade de São Paulo, Departamento de Saúde Ambiental, Faculdade de Saúde Pública - São Paulo - SP - Brazil.

**Keywords:** Nursing, Team, Occupational Health, Sleepiness, Working Conditions

## Abstract

**Objectives:**

To identify the factors associated with the occurrence of sleepiness among nursing professionals.

**Material and Methods:**

A case-control study nested in a cross-sectional one, conducted with 364 controls and 121 cases. Data on demographic and occupational characteristics were collected, as well as about lifestyle, physical and psychosocial work environment and somnolence. A multiple logistic regression analysis was performed.

**Results:**

The independent factors associated with the presence of sleepiness were as follows: imbalance between efforts and rewards (ORa=3.81; *p*<0.001), high overcommitment (ORa=3.20; *p*<0.001), workload equal to or greater than 45 hours a week (ORa=2.30; *p*=0.001), situations that can generate pain/injury with moderate or high exposure (ORa=1.85; *p*=0.037), and night work (ORa=1.71; *p*=0.038). The model was adjusted by gender and age group.

**Conclusion:**

Individual and historical-occupational characteristics and, mainly, those related to the physical and psychosocial work environment, were associated with the occurrence of sleepiness. Public and institutional preventive policies must include improvements in the conditions of the physical and psychosocial work environment, as well as strengthening of the individual resources.

## INTRODUCTION

In Brazil, there are more than 2.4 million nursing professionals active and working in assistance, managerial, and teaching/research areas^1^. The working conditions in the nursing context are characterized by intense physical and mental burdens. In recent years, work intensification due to the increase in the demands for quality of care, safety, productivity, precariousness of the labor relations, violence at work, incorporation of new technologies, epidemiological and demographic changes of the population, have been increasing unhealthy outcomes among workers^2^.

Fatigue, sleep deprivation and sleepiness exert direct impact on health and safety of nursing professionals, in addition to implications for quality of care and safety, early work disability, increased turnover, layoffs, and disability legal costs^3-6^. In China, 45.9% of the nursing professionals while working in hospitals reported the occurrence of avoidable errors due to medication administration, incorrect operation of equipment, needle injuries and falls of patients. Excessive sleepiness increases nearly 83% the chances for the occurrence of one of these events^7^.

Sleep disturbances often causes sleepiness that may have negative effects on daytime functioning and may results in health problems, impaired performance, and accidents^8,9^. Sleepiness refers to urge or tendency to fall asleep, difficulties to keep eyes open, unintentional napping, maintain concentration, and performance lapses besides including intrusions of sleeplike patterns into electrophysiological exams^10^.

The prevalence of sleepiness in nursing professionals worldwide varies between 2.8% in India^11^ and 53% in Nigeria^12^. Excessive somnolence can be the result of a chronic condition of difficulty initiating and maintaining sleep, in addition to a reduction in its duration due to conflicting circadian rhythms, particularly the sleep-wake cycle^13^. It is known that men, older people, and married individuals present higher chances of suffering from sleepiness^7,11,14-16^. In addition to that, aspects related to the working conditions, such as those arising from occupational exposure to psychosocial stressors and work organization^11,14,16^, are also involved in the occurrence of this phenomenon^16-19^. Nursing studies evaluating sleep disorders, fatigue or insomnia have already been published in the literature; however, few of them specifically assessed the issue of sleepiness and the work factors associated with it.

The promotion of decent work and healthy environment is part of the sustainable development goals (SDG) proposed by the United Nations (UN) for 2030^20^, which is also included in the Brazilian National Occupational Health Policy (*Política Nacional de Saúde do Trabalhador e Trabalhadora - PNSTT*)^21^. Considering the significant number of healthcare workers in the Brazilian national context and the negative impacts of work on health, this study can contribute to the SDG through the identification of work situations associated with sleepiness among nursing professionals.

## MATERIAL AND METHODS

A cross-sectional study was conducted among nursing professionals of São Paulo state (25% of contingent in Brazil), registered in 14 regional subsections of the Nursing Regional Council (Coren-SP) most of them in the São Paulo (35.0%) and Campinas (12.2%) subsections. Of the 411,162 professionals eligible, 1.0% (3,993 volunteers) enrolled in the study. Of this group, 942 (23.6%) were currently not practicing in the profession, while 3,051 (76.4%) were in active service, giving an overall enrolment rate of 0.74%. The main objective of the cross-s ectional study was to identify the profile of work ability (WA) and intention to leave the profession (ILP), in addition to describing individual characteristics and stressors of the physical and psychosocial work environment of this workforce. Details of this study can be seen elsewhere^22^. Among the participants of the cross-sectional study, 45.7% reported fair or bad sleep quality and 62.8% presented insomnia symptoms.

In order to identify work situations associated with sleepiness among nursing professionals we decided to conduct a nested case-control study. The sample size for this second study was calculated assuming the 52.0% frequency of the outcome of interest (occurrence of sleepiness)^23^, a=5.0% and (1-0)=90.0%, and a 20.0% replacement rate.

The sleepiness outcome was assessed by means of the Karolinska sleep questionnaire (KSQ) for the assessment of sleep quality^8^. The scale consists of 6 questions that address the following aspects: (1) sleepiness while at work, (2) sleepiness during off-work; (3) mental fatigue; (4) unintentional naps while at work; (5) unintentional naps during rest periods, and (6) fighting sleep to stay awake. The questions’ scores range from 1 to 5 points, where higher values mean higher sleepiness. The case are people with sleepiness (KSQ>3)^8^. We randomly select 3 people without sleepiness (controls: KSQ<4) for every 1 with sleepiness. The final sample was 121 individuals with sleepiness and 364 without sleepiness.

The data were collected online between October 2018 and March 2019. An electronic message was sent with access to the form containing questions about sociodemographic characteristics (gender, age, marital status, monthly family income and municipality of residence), lifestyle (smoking, CAGE questionnaire to assess risky alcohol consumption^24^, practice of physical activity and body mass index), occupational history (professional category, nursing education, age at entrance into workforce, years working in Nursing, main employment status, main area of performance, job position/function, second job, night shift, weekly working hours and recent history of workrelated disease or work injury).

To assess the working conditions, we evaluated the psychosocial work environment. Participants answered the following questionnaires:

The job stress scale (JSS), which is an adapted version for use in Brazil of the job content questionnaire, based on the demand-control model. It evaluates work demands, control and social support at work^25,26^. The ratio between demand and control was estimated, providing a score of 0.21 to 3.33 points, later categorized by tertiles, where the higher the score, the greater the risk of job strain^27-29^;

The psychosocial environment was also assessed using the adapted version for Brazilian version of the effort-reward imbalance (ERI) questionnaire, structured on the theoretical model of the same name, and consisting of the effort, reward and overcommitment dimensions^30,31^. The ratio between effort and reward was calculated and multiplied by 6/11, yielding a coefficient of 0.17 to 5.00 points, where scores over 1.0 indicate imbalance^30,31^;

The working conditions that lead to musculoskeletal disorders were assessed using the validated version for use in Brazil of the work-related activities that may contribute to pain and/or injury (WRAPI) questionnaire. It comprises 15 questions that provide a score from 0 to 150 points, where the higher the score, the worse the situation^32^. The scores were categorized in tertiles^29^.

All the scales got adequate reliability (Cronbach’s alpha coefficient >0.75), except for the JSS control scale (alpha=0.59), although it was maintained in the analyses given its relevance to the topic.

The association analysis was performed using the chisquare test and univariate and multiple logistic regression models. The models’ fit was assessed using the Hosmer-Lemeshow test. The risk measure was odds ratio (OR) and a descriptive *p*<0.050 level was used in all the analyses.

The study was approved by the Coren-SP board and by the Ethics and Research Committee of the School of Public Health, University of São Paulo (No. 2,614,513). The researchers did not have access to the professionals’ registration databases, in order to guarantee Coren-SP’s information safety premises (non-vulnerability and confidentiality). The workers’ participation was made effective by signing the free and informed consent form, and confidentiality of the individuals’ data was ensured. The study followed the principles set forth in the declaration of Helsinki and those of the Council of International Medical Sciences Organizations.

## RESULTS

Tables 1 and 2 present the analysis of the association between the occurrence of sleepiness and: a) the demographic and lifestyle characteristics (Table 1); and b) the occupational characteristics (Table 2). There was a statistically significant association between sleepiness and male gender (*p*=0.034), sedentary lifestyle (*p*=0.031), and overweight/obesity (*p*=0.039). The occupational variables/categories associated with the occurrence of sleepiness were as follows: areas of hospital and emergency care (*p*=0.021), function/position of providing direct care to the patient and head/leadership at an intermediate level (*p*=0.026), having a second job (*p*<0.001), night shift (*p*<0.001), weekly workload greater than or equal to 45 hours (*p*<0.001), and history of work-related injury or work- related disease (*p*<0.001) (Table 2).

**Table 1:** Distribution of nursing workers according to sleepiness, demographic and lifestyle characteristics. São Paulo State, Brazil, 2019 (n=364).

Characteristics	No sleepiness		Sleepiness		Total		p*
	**n**	**%**	**n**	**%**	**n**	**%**	
**Sex**							0.034
Female	315	76.8	95	23.2	410	100.0	
Male	49	65.3	26	34.7	75	100.0	
**Age (Years)**							0.685
Up to 30.9	56	77.8	16	22.2	72	100.0	
31.0 to 40.9	121	72.9	45	27.1	16	100.0	
41.0 and more	187	75.7	60	24.3	247	100.0	
**Marital status**							0.563
Married/living with a partner	211	75.6	68	24.4	279	100.0	
Divorced/widowed	49	70.0	21	30.0	70	100.0	
Single	104	76.5	32	23.5	136	100.0	
**Monthly family income (national minimum wages)**							0.709
Up to 3.0	30	27.3	8	7.3	110	100.0	
3.1 to 5.0	70	52.2	17	12.7	134	100.0	
5.1 to 7.0	59	72.8	23	28.4	81	100.0	
7,1 to 10.0	97	114.1	33	38.8	85	100.0	
10.1 and more	99	230.2	38	88.4	43	100.0	
I don’t want to answer	9	75.0	2	16.7	12	100.0	
**Place of residence**							0.234
Capital	133	78.2	37	21.8	170	100.0	
Countryside	231	73.3	84	26.7	315	100.0	
**Human development Index**							0.546
Very high	201	76.1	63	23.9	264	100.0	
High	163	73.8	58	26.2	221	100.0	
**Smoking**							0.294
Never smoked	251	74.0	88	26.0	339	100.0	
Former smoker	67	73.6	24	26.4	91	100.0	
Current smoker	46	83.6	9	16.4	55	100.0	
**Alcohol use risk**							0.811
No	345	75.2	114	24.8	459	100.0	
Yes	19	73.1	7	26.9	26	100.0	
**Regular practice of physical activity**							0.031
Yes	164	80.0	41	20.0	205	100.0	
No	200	71.4	80	28.6	280	100.0	
**Body mass index**							0.039
Normal	123	82.0	27	18.0	150	100.0	
Overweight	124	73.4	45	26.6	169	100.0	
Obesity	112	69.6	49	30.4	161	100.0	
Not informed	5	100.0	0	0.0	5	100.0	
**Total**	**364**	**75.1**	**121**	**24.9**	**485**	**100.0**	

Note:

* Chi-square test.

**Table 2:** Distribution of nursing workers according to sleepiness and occupational characteristics. São Paulo State, Brazil, 2019 (n=364).

Characteristics	No sleepiness		Sleepiness		Total		p*
	**n**	**%**	**n**	**%**	**n**	**%**	
**Professional category**							0.053
Registered nurse	227	78.5	62	21.5	289	100.0	
Nurse technician	110	68.3	51	31.7	161	100.0	
Nurse assistant	27	77.1	8	22.9	35	100.0	
**Nursing education**							0.109
Postgraduate degree	177	78.7	48	21.3	225	100.0	
College education	50	78.1	14	21.9	64	100.0	
High and elementary school	137	69.9	59	30.1	196	100.0	
**Age at entrance into the workforce (years)**							0.465
18.0 and more	174	75.3	57	24.7	231	100.0	
14.0 to 17.9	153	76.5	47	23.5	200	100.0	
Up to 14.0	37	68.5	17	31.5	54	100.0	
**Time in the nursing profession**							0.719
Up to 6.0	56	78.9	15	21.1	71	100.0	
6.0 to 10.9	71	74.0	25	26.0	96	100.0	
11.0 to 15.9	62	71.3	25	28.7	87	100.0	
16.0 and more	175	75.8	56	24.2	231	100.0	
**Main employment contract**							0.146
Formal contract in a private institution	181	72.1	70	27.9	251	100.0	
Civil servant	135	76.3	42	23.7	177	100.0	
Others	48	84.2	9	15.8	57	100.0	
**Main area of performance**							0.021
Hospital	168	71.8	66	28.2	234	100.0	
Primary health care	87	80.6	21	19.4	108	100.0	
Emergence services	34	64.2	19	35.8	53	100.0	
Others	75	83.3	15	16.7	90	100.0	
**Position/function**							0.026
Direct patient care	248	71.9	97	28.1	345	100.0	
Headship/Leadership at operational level	49	74.2	17	25.8	66	100.0	
Headship/Leadership at corporate level	12	100.0	0	0.0	12	100.0	
Advice/Consulting/Specialist	20	87.0	3	13.0	23	100.0	
Teaching/Research	12	85.7	2	14.3	14	100.0	
Others	23	92.0	2	8.0	25	100.0	
**Second job**							<0.001
No	253	82.7	53	17.3	306	100.0	
yes	111	62.0	68	38.0	179	100.0	
**Working at night shift (1^st^ and/or 2^nd^ job)**							<0.001**
No	270	79.9	68	20.1	338	100.0	
Yes	94	63.9	53	36.1	147	100.0	
**Weekly working hours**							<0.001
Up to 39.9	144	79.1	38	20.9	182	100.0	
40.0 to 49.9	127	83.6	25	16.4	152	100.0	
45.0 and more	93	61.6	58	38.4	151	100.0	
**Work-related disease or injury** No	263	81.4	60	18.6	323	100.0	<0.001
Yes	101	62.3	61	37.7	162	100.0	
**Total**	**364**	**75.1**	**121**	**24.9**	**485**	**100.0**	

Note:

* Chi-square test.

All job characteristics (Table 3) presented a statistically significant association: a higher risk for sleepiness was observed in high work demand (*p*<0.001), low control over work (*p*=0.028), low social support at work (*p*<0.001), moderate or high demand/ control ratio (*p*<0.001), high effort at work (*p*<0.001), low rewards at work (*p*<0.001), high overcommitment (*p*<0.001), effort/reward imbalance (*p*<0.001), and moderate and high exposure to situations that favor pain or injury (*p*<0.001).

**Table 3:** Distribution of nursing workers according to sleepiness and working conditions. São Paulo State, Brazil, 2019 (n=364).

Characteristics	No sleepiness		Sleepiness		Total		p*
	**n**	**%**	**n**	**%**	**n**	**%**	
**Demands at work**							<0.001
Lower	61	92.4	5	7.6	66	100.0	
High	303	72.3	116	27.7	419	100.0	
**Control at work**							0.028
High	283	77.5	82	22.5	365	100.0	
Low	81	67.5	39	32.5	120	100.0	
**Social support at work**							<0.001
High	300	78.7	81	21.3	381	100.0	
Low	64	61.5	40	38.5	104	100.0	
**Demand/control ratio**							<0.001
Low	126	87.5	18	12.5	144	100.0	
Moderate	90	78.9	24	21.1	114	100.0	
High	148	65.2	79	34.8	227	100.0	
**Efforts at work**							<0.001
Low	287	82.9	59	17.1	346	100.0	
High	77	55.4	62	44.6	139	100.0	
**Rewards at work**							<0.001
High	309	82.4	66	17.6	375	100.0	
Low	55	50.0	55	50.0	110	100.0	
**Overcommitment**							<0.001
Low	215	87.8	30	12.2	245	100.0	
High	149	62.1	91	37.9	240	100.0	
**Effort-reward imbalance**							<0.001
No	323	82.6	68	17.4	391	100.0	
Yes	41	43.6	53	56.4	94	100.0	
**Work-related activities that lead do pain and/or injury**							<0.001
Low	144	87.8	20	12.2	164	100.0	
Moderate	99	68.3	46	31.7	145	100.0	
High	121	68.8	55	31.3	176	100.0	
**Total**	**364**	**75.1**	**121**	**24.9**	**485**	**100.0**	

Note:

* Chi-square test.

The multiple logistic regression analysis (Table 4) showed the independent variables statistically associated with the occurrence of sleepiness were the following: imbalance between efforts and rewards (ORa=3.81; *p*<0.001), high overcommitment (ORa=3.20; *p*<0.001), working time equal to or greater than 45 hours a week (ORa=2.30; *p*=0.001), situations that can generate pain/injury with moderate or high exposure (ORa=1.85; *p*=0.037), and night work (OR=1.71; *p*=0.038). The model was adjusted for gender and age group, and the HosmerLemeshow residual analysis (x^2^=10.75; *p*=0.216) showed good fit of the model.

**Table 4:** Multiple logistic regression analysis of factors associated with sleepiness among nursing professionals. São Paulo State, Brazil, 2019 (n=364).

Variables	OR_adjust_	95%CI Inf.	95%CI Sup.	p
**Effort-reward imbalance** No	>1.00			
Yes	3.81	2.22	6.54	<0.001
**Overcommitment** Low	>1.00			
High	3.20	1.90	5.40	<0.001
**Weekly working hours** Up to 49.9	>1.00			
45.0 and more	2.30	1.39	3.80	0.001
**Work-related activities that lead do pain and/or injury** Low	>1.00			
Moderate/high	1.85	1.04	3.28	0.037
**Working at night shift (1^st^ and/or 2^nd^ job)** No	>1.00			
Yes	1.72	1.03	2.87	0.038
**Sex** Female	>1.00			
Male	1.73	0.92	3.23	0.087
**Age (Years)** Up to 40.9	>1.00			
41.0 and more	0.93	0.62	1.40	0.733

## DISCUSSION

This study evaluated the possible association of sociodemographic, occupational and lifestyle variables and environmental and psychosocial working conditions with the occurrence of sleepiness among nursing professionals. It was observed that the outcome was explained by imbalance between efforts and rewards, high overcommitment, high workload, moderate or high number of situations that can contribute to musculoskeletal pain or injury, and night work.

The psychosocial factors at work considered negative for the workers’ health were associated with sleepiness. The perception of imbalance between efforts while performing work and the corresponding rewards increased almost four times the outcome probability. Situations of imbalance between efforts and rewards are frequent in nursing and are associated with an increased risk of physical and mental health impairments; quality and duration of sleep plays a mediating role in the health of these workers^33^. In a systematic review by Linton et al. (2015)^34^, it was observed that a job with such characteristics represented a risk for sleep disorders, such as insomnia, which was found among industrial workers in Japan^17,35^. The imbalance between efforts and rewards is a determinant of stress at work^36^ and, therefore, it can affect the hypothalamic-pituitaryadrenocortical and sympathetic-adrenomedullary systems, favoring the occurrence of insomnia^17^ and this, in turn, would be associated with sleepines^37^.

Overcommitment to work, recognized as an intrinsic effort by the worker, increased more than three times the risk of sleepiness. A Swedish study indicated that aspects such as satisfaction regarding the workload and time to perform the tasks, as well as satisfaction with the time for rest-recoverysleep are associated with the outcome^16^. Overcommitment is frequent in nursing and is associated with an increased risk for stress and showed a negative impact on work ability^29^. It can be explained, as workers tend to underestimate the work demands and overestimate their coping resources, potentiating the detrimental effects, with increased susceptibility to burnout and exhaustion^36^. Exaggerated dedication to work is associated with insomnia^35^ and the authors suggest a sequence that involves apprehension in relation to the subsequent workday and reduction in sleep duration.

A weekly workload equal to or greater than 45 hours increased 2-3 times the chance of sleepiness. An 8-hour/day workday increased the chance of sleepiness both in a study conducted in Thailand^14^ and in India^11^. The systematic review by Linton et al. (2015)^34^ was not able to determine the effect of workload on sleep disorders, as this condition can be evaluated using several methods, which may show distinct outcomes. Longer workdays can be more stressful than shorter ones due to longer interactions between the professionals and the harmful context, leading to negative repercussions on sleep. Work regimes with long working hours are frequent in contemporary society included in health services. They increase the risk for the occurrence of sleep disorders, which in turn is associated with threats to workers’ health, such as cardiovascular diseases, mental disorders, impairment of cognitive functions and accidents, in addition to exhaustion and fatigue^4,38^.

The night work shift increased the chance of sleepiness and was also associated in a study conducted in Greece^39^ and in another carried out in China^7^ with nursing professionals. This result was not found in Thailand^14^, where working-rotating shifts was more detrimental that fixed shifts. In Norway, the work shift was not associated with the outcome, but there was a decrease in sleepiness among those who left the night shift for the day shift when compared to those who stayed on the night shift^40^. During night work, the circadian process of the sleepwake cycle, which involves increased pressure to fall asleep, gains relevance, causing difficulties to stay awake, resulting excessive sleepiness and fatigue throughout the night shift, with progressive difficulty in performing the work activities^3^. There is also a mediating effect of sleep on the physical and mental health of the nursing professionals who work in the night shift^33^.

The nursing professionals’ work activities routines, such as moving patients in bed, supporting patients to move between a bed and a chair, lifting heavy loads and physical resistance during work are frequently reported^29,41^. These conditions are among the main causes of the high occurrence of musculoskeletal disorders in this workforce^29,41^. Although a Norwegian study did not find any association between physical workloads and sleep disorders^42^, a work situation with moderate or high exposure that can generate pain or musculoskeletal injury increases by 85% the chance of sleepiness as observed in this Brazilian study. An observational study with nursing professionals in Turkey showed that the severity of insomnia was significantly higher among those with musculoskeletal problems, possibly because the pain resulting from these disorders can cause sleep disorders^41^. It is likely that this relationship is mediated by the same hyperactivity component of the sympathetic system and its clinical consequences.

The literature data are controversial regarding the association between sleepiness and gender. Some studies indicate an association with women^15^, others with men^11^ and, for a third group of studies, there is no statistically significant association^7,39^. The age variable was also not statistically significant, a fact similar to studies conducted in several places of the world^7,12,39,43^. In the present study, this variable was not associated with a higher probability of sleepiness; however, it was included as an adjustment of the final model.

The results showed that the working conditions play a central role in the occurrence of sleepiness among nursing professionals. Practical recommendations can be targeted based on the results observed. Actions aimed at work organization seeking to balance efforts and rewards are a priority, as well as ergonomic interventions to correct or reduce work demands that involve physical loads, and adequacy of activity requirements to the duration of the work shift and the available staffing; in addition to institutional actions that seek to support the workers, avoiding situations that favor overcommitment trends, as well as actions to encourage the professionals to recognize, report and protect themselves against situations that favor fatigue. Work schedule arrangements that include long hours and repetition of consecutive night shifts must be avoided; and appropriate times and places for rest should be planned during shift workdays4,6,33,44.

These actions are favored by adequate public policies regulating working hours and minimum wages, in order to avoid long working times, two jobs, favoring rest and recovery periods between shifts. Institutional actions aimed at ergonomic improvements are also necessary, as well as related to people management and assistance practices. In addition to that, inspection and education actions by nursing professional bodies and strengthening of surveillance in occupational health and assessment of work injuries, adverse events and risk situations can also provide subsidies for targeting interventions. It is to be noted that the improvement of the working conditions would not only favor the prevention of sleepiness with reduction of the risks to the workers’ health and safety and quality of care for the patients/ clients, but also are the basis for the prevention of various negative outcomes and the promotion of worker’s health^4,6,33,44^.

### Limitations and strengths

The study presents external validity as it sampled the population of nursing professionals from the most populous state of Brazil, who carry out their work activities in different places and with different complexity levels. The differential of this study is that it is one of the few that specifically addresses the issue of sleepiness and the work factors associated with it and, in this way, the results can support preventive actions and health promotion for workers and improve the quality of care for patients.

Sleep quality during working times and off-work were not recorded; neither other important chronobiological parameters that are associated with sleepiness, such as habits and preferences (e.g., time to go to bed and wake up during free days, time to perform activities at work and off-work).

The inclusion of professionals working in several places can lead to a bias resulting from different working conditions and population contexts. To minimize the analysis bias, we evaluated non-occupational factors, such as sociodemographic and lifestyle characteristics, as well as it contemplated the different professional nursing categories and their areas of work activities, but at the final analysis, these features were not the most important. As this is not a longitudinal study, it was not possible to assess causal relationships between the independent variables and sleepiness in the study population.

## CONCLUSION

This study showed that psychosocial factors at work like effort-reward imbalance and high overcommitment to work, weekly working time 45 hours or more, night shift and tasks that can generate pain/injury with moderate or high exposure were independently associated with the occurrence of sleepiness among nursing professionals.

Public and institutional policies should include actions to ensure better work environment, as well as interventions to improve shift schedules, intensify occupational surveillance and strengthen individual resources. Such actions would not only favor the prevention of sleepiness reducing the risks to the workers’ health and to the quality and safety care for the patients and clients but are also the basis for prevention of several negative outcomes and for promotion of workers’ health.

## References

[1] Conselho Regional de Enfermagem de São Paulo (Coren) (2021). Institucional [Internet].

[2] Martinez MC, Fischer FM (2019). Work ability as determinant of termination of employment: to resign or be dismissed?. J Occup Environ Med.

[3] Wilson M, Permito R, English A, Albritton S, Coogle C, Van Dongen HPA (2019). Performance and sleepiness in nurses working 12-h day shifts or night shifts in a community hospital. Accid Anal Prev.

[4] Hãrmã M, Koskinen A, Sallinen M, Kubo T, Ropponen A, Lombardi DA (2020). Characteristics of working hours and the risk of occupational injuries among hospital employees: a case-crossover study. Scand J Work Environ Health.

[5] James L, James SM, Wilson M, Brown N, Dotson EJ, Dan Edwards C (2020). Sleep health and predicted cognitive effectiveness of nurses working 12hour shifts: an observational study. Int J Nurs Stud.

[6] American Nurses Association (ANA) (2014). Position paper: addressing nurse fatigue to promote safety and health: joint responsibilities of registered nurses and employers to reduce risks [Internet].

[7] Chen L, Luo C, Liu S, Chen W, Liu Y, Li Y (2019). Excessive daytime sleepiness in general hospital nurses: prevalence, correlates, and its association with adverse events. Sleep Breath.

[8] Nordin M, Âkerstedt T, Nordin S (2013). Psychometric evaluation and normative data for the Karolinska Sleep Questionnaire. Sleep Biol Rhythms.

[9] Âkerstedt T, Garefelt J, Richter A, Westerlund H, Hanson LLM, Sverke M (2015). Work and sleep - a prospective study of psychosocial work factors, physical work factors, and work scheduling. Sleep.

[10] Âkerstedt T (1988). Sleepiness as a consequence of shift work. Sleep.

[11] Mathew JJ, Joseph M, Britto M, Joseph B (2018). Shift work disorder and its related factors among health-care workers in a tertiary care hospital in Bangalore, India. Pakistan J Med Sci.

[12] Aliyu I, Ibrahim Z, Teslim L, Okhiwu H, Peter I, Michael G (2017). Sleep quality among nurses in a tertiary hospital in North-West Nigeria YR - 2017/7/1. Niger Postgrad Med J.

[13] Akerstedt T (2003). Shift work and disturbed sleep/wakefulness. Occup Med (Lond).

[14] Chaiard J, Deeluea J, Suksatit B, Songkham W, Inta N (2018). Short sleep duration among Thai nurses: influences on fatigue, daytime sleepiness, and occupational errors. J Occup Health.

[15] D’ettorre G, Pellicani V, Caroli A, Greco M (2020). Shift work sleep disorder and job stress in shift nurses: Implications for preventive interventions. Med Lav.

[16] Magnusson L, Hâkansson C, Brandt S, Oberg M, Orban K (2020). Occupational balance and sleep among women. Scand J Occup Ther.

[17] Ota A, Masue T, Yasuda N, Tsutsumi A, Mino Y, Ohara H (2009). Psychosocial job characteristics and insomnia: a prospective cohort study using the demand-control-support (DCS) and effort-reward imbalance (ERI) job stress models. Sleep Med.

[18] Âkerstedt T, Garefelt J, Richter A, Westerlund H, Hanson LLM, Sverke M (2015). Work and sleep—a prospective study of psychosocial work factors, physical work factors, and work scheduling. Sleep.

[19] Portela LF, Luna CK, Rotenberg L, Silva-Costa A, Toivanen S, Araújo T (2015). Job strain and self-reported insomnia symptoms among nurses: what about the influence of emotional demands and social support?. Biomed Res Int.

[20] Organização das Nações Unidas - Brasil (ONU) Objetivos de desenvolvimento sustentável [Internet].

[21] Ministério da Saúde (BR) (2012). Portaria n° 1.823, de 23 de agosto de 2012. Institui a Política Nacional de Saúde do Trabalhador e da Trabalhadora.

[22] Martinez MC, Latorre MRDO, Fischer FM (2021). Capacidade para o trabalho e intenção de saída da profissão na enfermagem de São Paulo. Rev Enferm UERJ.

[23] Silva FJ, Felli VEA, Martinez MC, Mininel VA, Ratier APP (2016). Association between work ability and fatigue in Brazilian nursing workers. Work.

[24] Marques ACPR, Ribeiro M, Laranjeira R, Oliveira R, Nobre MR, Bernardo WM (2003). Usuários de substâncias psicoativas: abordagem, diagnóstico e tratamento.

[25] Karasek R, Brisson C, Kawakami N, Houtman I, Bongers P, Amick B (1998). the job content questionnaire (JCQ): an instrument for internationally comparative assessments of psychosocial job characteristics. J Occup Health Psychol.

[26] Alves MGM, Chor D, Faerstein E, Lopes CS, Werneck GL (2004). Short version of the “job stress scale”: a Portuguese-language adaptation. Rev Saúde Pública.

[27] Theorell T, Perski A, Akerstedt T, Sigala F, Ahlberg-Hulten G, Svensson J (1988). Changes in job strain in relation to changes in physiological state. A longitudinal study. Scand J Work Environ Health.

[28] Landsbergis PA, Schnall PL, Warren K, Pickering TG, Schwartz JE (1994). Association between ambulatory blood pressure and alternative formulations of job strain. Scand J Work Environ Health.

[29] Martinez MC, Latorre MRDO, Fischer FM (2015). A cohort study of psychosocial work stressors on work ability among Brazilian hospital workers. Am J Ind Med.

[30] Chor D, Werneck GL, Faerstein E, Alves MGM, Rotenberg L (2008). The Brazilian version of the effort-reward imbalance questionnaire to assess job stress. Cad Saúde Pública.

[31] Siegrist J (2008). Effort-reward imbalance and health in a globalized economy. SJWEH Suppl.

[32] Coluci MZO, Alexandre NMC (2009). Adaptação cultural de instrumento que avalia atividades do trabalho e sua relação com sintomas osteomusculares. Acta Paul Enferm.

[33] Hasselhorn HM, Müller BH, Tackenberg P (2005). NEXT scientific report - July 2005.

[34] Linton SJ, Kecklund G, Franklin KA, Leissner LC, Sivertsen B, Lindberg E (2015). The effect of the work environment on future sleep disturbances: a systematic review. Sleep Med Rev.

[35] Ota A, Masue T, Yasuda N, Tsutsumi A, Mino Y, Ohara H (2005). Association between psychosocial job characteristics and insomnia: an investigation using two relevant job stress models - The demand-control-support (DCS) model and the effort-reward imbalance (ERI) model. Sleep Med.

[36] Siegrist J (2005). Social reciprocity and health: New scientific evidence and policy implications. Psychoneuroendocrinology.

[37] Wilsmore BR, Grunstein RR, Fransen M, Woodward M, Norton R, Ameratunga S (2013). Sleep habits, insomnia, and daytime sleepiness in a large and healthy community-based sample of New Zealanders. J Clin Sleep Med.

[38] Wong K, Chan AHS, Ngan SC (2019). The effect of long working hours and overtime on occupational health: a meta-analysis of evidence from 1998 to 2018. Int J Environ Res Public Health.

[39] Alexandropoulou A, Vavougios GD, Hatzoglou C, Gourgoulianis KI, Zarogiannis SG (2019). Risk assessment for self-reported obstructive sleep apnea and excessive daytime sleepiness in a Greek nursing staff population. Medicina (Kaunas).

[40] Thun E, Bjorvatn B, Äkerstedt T, Moen BE, Waage S, Molde H (2016). Trajectories of sleepiness and insomnia symptoms in Norwegian nurses with and without night work and rotational work. Chronobiol Int.

[41] Cici R, Yilmazel G (2021). Musculoskeletal disorders increases the insomnia severity in nurses. Sleep Sci.

[42] Eriksen W, Bjorvatn B, Bruusgaard D, Knardahl S (2008). Work factors as predictors of poor sleep in nurses’ aides. Int Arch Occup Environ Health.

[43] Saksvik-Lehouillier I, Bjorvatn B, Mageroy N, Pallesen S (2016). Hardiness, psychosocial factors and shift work tolerance among nurses - a 2-year follow-up study. J Adv Nurs.

[44] Dall’Ora C, Dahlgren A (2020). Shift work in nursing: closing the knowledge gaps and advancing innovation in practice. Int J Nurs Stud.

